# Respiratory Illness-related Emergency Visits Among Children, COVID-19 and Beyond: Observing a Return to Seasonal Patterns?

**DOI:** 10.5811/westjem.46552

**Published:** 2025-12-20

**Authors:** Mamata V. Kene, Madeline J. Somers, Dustin W. Ballard, Dana R. Sax, Mary E. Reed, Tara L. Greenhow

**Affiliations:** * The Permanente Medical Group, Oakland, California; † Kaiser Foundation Hospital, Department of Emergency Medicine, Fremont, California; ‡ Kaiser Permanente Division of Research, Pleasanton, California; § Kaiser Foundation Hospital, Department of Emergency Medicine, Oakland, California; || Kaiser Foundation Hospital, Department of Pediatric Infectious Diseases, San Francisco, California; # Kaiser Foundation Hospital, Department of Emergency Medicine, San Rafael, California

## Abstract

**Introduction:**

The COVID-19 pandemic disrupted care-seeking and respiratory disease epidemiology across healthcare settings, notably for emergency department (ED) care. The scope of this disruption and whether patterns of ED visits have returned to predictable seasonal patterns is of interest in planning ED staffing and resource availability for future illness surges, pandemic or not. We evaluated ED visits for acute respiratory illness among children in a large, integrated healthcare delivery system to describe illness and patient characteristics in the years before, during, and after the pandemic peak.

**Methods:**

We conducted a cross-sectional study of ED visits among patients 0–17 years of age to the 21 EDs of Kaiser Permanente Northern California, from January 1, 2018–December 31, 2019, pre-pandemic; January 1, 2020–December 31, 2021, pandemic; and January 1, 2022–March 31, 2024, post-vaccine (vaccines for children > 5 years of age approved and available). We electronically extracted eligible ED visits with acute respiratory infection diagnoses and a range of sociodemographic, medical comorbidity, and utilization characteristics.

**Results:**

We observed 151,983 pediatric ED visits with eligible respiratory infection diagnoses, 49,912 (32.8%) visits pre-pandemic, 27,109 (17.8%) visits during the pandemic, and 74,962 (49.3%) visits post-vaccine. Eligible visits dropped every month from 6,361 in February 2020, just prior to the pandemic onset, to their lowest volume (243) in June 2020. In the post-vaccine period, visits peaked at 10,638 in November 2022, the highest of any month during the study period. Sex, race/ethnicity, and tobacco exposure were comparable over time, but the proportion of visits by patients with under-immunized diagnosis trended upward over time. Upper respiratory infection (30% pre-pandemic, 32% pandemic, and 33% post-vaccine periods), asthma (15% pre-pandemic, 12% pandemic, and 12% post-vaccine periods), and cough (9.9% pre-pandemic, 12% pandemic, and 12% post-vaccine periods), were the top three diagnoses across all periods.

**Conclusion:**

In this cross-sectional study of acute respiratory illness-related ED visits in an integrated healthcare system, from 2022 onward seasonal variation in respiratory illness ED visits rebounded, with notable and unseasonal peaks in late 2022. COVID-19 appears to be a minor contributor to ED visits for pediatric respiratory illness. However, an increased overall and seasonal burden of ED visits has implications for surge planning and mitigation, with COVID-19 now being endemic and typical respiratory pathogens having resurfaced.

## INTRODUCTION

The COVID-19 pandemic changed the epidemiology of respiratory illnesses with repercussions for emergency care and preparedness. Among children, this impact was particularly notable, as respiratory infections are the top reason for pediatric emergency department (ED) visits.^1^ Early pandemic COVID-19 mitigation strategies were associated with a dramatic decrease in non-COVID-19 viral respiratory infections that later rebounded.^2^,^3^ Pediatric ED visits for acute respiratory illnesses followed a similar pattern.^4^–^6^ With the return to previous activities including school attendance and travel, acute respiratory illnesses would be expected to rebound. But immunity gaps from decreased exposure to circulating viral pathogens and disrupted seasonal patterns of endemic respiratory viruses may also impact the severity, timing, and age distribution of acute respiratory illnesses and ED visits for these conditions.^7^–^10^ The fall 2022 respiratory syncytial virus (RSV) surge observed in the Northern Hemisphere was such an event, straining healthcare systems because of the severity and volume of illness.^11^–^13^ Less is reported about the broader landscape of acute respiratory illness among children as the pandemic has become endemic, yet as ED leaders plan staffing and resource allocation, understanding whether and how ED visits for these conditions returned to pre-pandemic patterns is important. We thus describe ED visits for acute respiratory illness among children in a large, integrated healthcare delivery system in the years before, during, and after the pandemic peak.

## METHODS

### Study Setting and Design

We conducted a retrospective, cross-sectional observational study of ED visits among patients 0–17 years of age to the 21 EDs of Kaiser Permanente Northern California (KPNC) from January 1, 2018–March 31, 2024. Kaiser Permanente Northern California is an integrated healthcare delivery system serving over four million members with over 1.2 million ED visits annually. The KPNC members are similar sociodemographically and in health status to the surrounding communities.^14^ The Kaiser Foundation Institutional Review Board approved the study protocol and waived the requirement for informed consent.

All data were electronically extracted from the electronic health record (EHR) (Epic Systems Corporation, Verona, WI) by an experienced programmer (MS); no manual chart review was conducted. All ED visits during the study period with an eligible acute respiratory illness diagnosis and health plan membership were electronically extracted. Acute respiratory illness ED visits were extracted based on *International Classification of Diseases, 10^th^ Modification* (*ICD-10*)-coded visit diagnoses (Appendix) based on the Pediatric Clinical Classification System (PE-CCS) (ref: https://www.childrenshospitals.org/content/analytics/toolkit/pediatric-clinical-classification-system-peccs-codes accessed March 4, 2025). To ensure complete data were available from the EHR, we restricted the study group to patients with health plan membership at the time of the visit if <12 months of age, and nine of the prior 12 months for patients >12 months of age. We extracted patient characteristics and comorbidities that may be associated with ED use and with respiratory illness. Because social determinants of health including race or ethnicity, neighborhood deprivation and insurance status are associated with ED use among children, we included these measures.^15^ Sociodemographic variables include age, sex, race/ethnicity, neighborhood deprivation index (a measure of socioeconomic status that includes income, housing quality, employment and education data points at the ZIP code level), and tobacco exposure.

**Population Health Research Capsule**

What do we already know about this issue?

*The COVID-10 pandemic broadly disrupted non-COVID-19 disease epidemiology and health care utilization. Seasonal disease surges, for which emergency departments plan staffing and resources, altered as well*.

What was the research question?

*How did respiratory disease emergency visits among pediatric patients alter during and after the COVID-19 pandemic?*

What was the major finding of the study?

*The early pandemic period saw a 91% decline in pediatric emergency visits for respiratory illness, with a slow rebound and resumption of seasonal patterns by late 2022*.

How does this improve population health?

*Disaster and surge planning for future pandemics and public health emergencies may take into account a protracted tail of impact on health care utilization as well as potential rebound surges in non-pandemic diseases*.

Medical comorbidities extracted were under-immunization *ICD-10* diagnosis and chronic conditions from the PE-CCS. Because under-immunized children have different patterns of healthcare use and chronic conditions are associated with increased ED use, we included these characteristics to describe the study cohort.^16^,^17^ All variables were electronically extracted from structured data fields in the EHR. The study adhered to components of best practices for data extraction defined by Worster et al (2005) including case selection criteria, clearly defined variables, and medical record identification.^18^

### Descriptive Statistics

We calculated ED visits by month for included diagnoses and for all pediatric ED visits. Absence of a comorbidity was considered not having that diagnosis. Missing data are acknowledged in the tables, but as no inferential analysis was conducted, no adjustments for missing data were made. We divided the study into three periods: January 1, 2018–December 31, 2019 (pre-pandemic); January 1, 2020–December 31, 2021 (pandemic); and January 1, 2022–March 31, 2024 (post-vaccine). Visits were calculated by month for all ED visits and for respiratory illness ED visits. We collated descriptive data for each of the three observation periods.

## RESULTS

After applying inclusion criteria, we observed 151,983 pediatric ED visits with eligible acute respiratory illness diagnoses. Patient characteristics are presented in Table 1; we noted 49,912 (32.8%) visits pre-pandemic; 27,109 (17.8%) visits during the pandemic period; and 74,962 (49.3%) visits post-vaccine. Sex, race/ethnicity, and tobacco exposure were comparable over time, but the proportion of visits by patients who were under-immunized trended upward over time.

The figure presents ED visits for all-cause, acute respiratory illness and COVID-19 illness during the study period by month. By quarter, pre-pandemic acute respiratory illness visits peaked in January–March 2019 at 15,959 visits. In the pandemic period, visits dropped to their lowest point in April–June 2020 (767 visits). At the pandemic onset, monthly visits dropped from 6,361 in February 2020 to 3,076 in March 2020 and further to 274 in April 2020, before reaching a nadir of 243 in June 2020.

For the winter quarter peak respiratory illness season (January–March), a nadir of 1,718 visits was reached in 2021, 11% of 2020 winter quarter volume. The following years’ winter quarters—2022 (6,897, fourfold increase from 2021), 2023 (10,324, 50% increase from 2022) and 2024 (12,337, 20% increase from 2023) —saw a gradual rebound in ED visits. The monthly peak in ED visits occurred in November 2022 with 10,638 visits, with fall (October–December) quarter 2022 seeing 25,363 visits compared to the years prior and after (9,667 and 14,272 visits in fall quarters 2021 and 2023, respectively). COVID-19 diagnoses accounted for a minority of visits from 2020–2024. In Table 2, upper respiratory infection (30% pre-pandemic, 32% pandemic and 33% post-vaccine); asthma (15% pre-pandemic and 12% pandemic and post-vaccine); and cough (9.9% pre-pandemic, 12% pandemic and 11% post-vaccine) were the most common visit diagnoses.

## DISCUSSION

In this cross-sectional study of acute respiratory illness-related ED visits in an integrated healthcare system, we noted a 91% decline in visits at pandemic onset (3,076 visits in March 2020 dropping to 274 visits in April 2020), followed by a trough and slow rebound, peaking at 10,638 visits in November 2022. Seasonal oscillation resumed in 2022 and continued into 2024. Patient characteristics were comparable across the study periods, with the three most common visit diagnoses (upper respiratory infection [30–33% of visits], asthma [12–15% of visits], and cough [9.9–12%]) stable across the three epochs.

Seasonal surges of respiratory illness before COVID-19 attenuated early in the pandemic, prompting uncertainty about the pandemic’s longer term impact on seasonal respiratory illness epidemiology and severity.^8^ A few studies reported rebounding respiratory infection ED visits and hospital admissions in 2021 after social distancing restrictions were lifted, school attendance and travel normalized, and COVID-19 immunizations were widely available,^19^,^20^ but little is reported beyond 2021. We included a broader list of acute respiratory illness to understand ED use and demand and extended our observations into 2024.

Like other studies, we report a 90% drop in ED visits early in the pandemic, from winter quarter to spring quarter 2020, notable even after considering that winter is the traditional peak in seasonal acute respiratory illnesses.^5^,^6^ However, seasonal fluctuations in monthly visits resumed in 2022. While pre-pandemic winter quarters (January–March) noted the highest visit volumes (15,000–16,000 visits), in the post-vaccine period, quarterly visits peaked in fall 2022 (> 25,000 visits), earlier in the fall/winter respiratory illness seasons than prior to the pandemic. The marked peak observed in fall 2022 may arise from immunity gaps attributable to decreased individual and population respiratory-pathogen exposure intersecting with a return to usual activities.^21^ While deferred healthcare-seeking might impact ED volumes, this effect might be more substantial for primary care visits, and we would expect the variation observed mostly reflects the prevalence of illness.

The noticeable dip in ED use during the pandemic has raised questions about future ED volumes and staffing needs. As overall ED visits rebounded in 2021–2022 (but not to pre-pandemic levels), speculation ensued about consequent decreased ED workforce requirements.^22^ This concern, however, may be premature if our observation of progressive increases in ED visits four years after the pandemic holds true for all-cause ED visits as well as respiratory illnesses. Furthermore, COVID-19-associated healthcare worker attrition may exacerbate staffing challenges in future pandemics and recovery periods.^23^ Finally, much disaster planning focuses on immediate response, but we report elevated ED volumes and notable monthly peaks up to four years after the pandemic onset. Future pandemic planning for ED staffing and resource allocation might consider possible monthly volatility and excess demand long after the acute pandemic phase.

Across the three observation periods, patients were comparable in age, race/ethnicity, socioeconomic status (Neighborhood Deprivation Index), and the frequency of chronic conditions. This finding likely reflects that no major changes in health plan membership occurred during the study period. Nationally, ED visits followed similar trends across age, race, and ethnicity groups.^24^ Tracking granular, stratified ED-visit patterns in future pandemics, disasters and recovery periods would promptly identify disproportionate effects on vulnerable groups. We did observe a trend of increased frequency of prior under-immunization diagnosis in the pandemic and post-vaccine periods. We could not ascertain whether this finding reflects missing or lagging COVID-19 vaccinations, influenza vaccine refusal, or a decline in routine childhood immunizations. Since a general decrease in immunization rates during the pandemic was also observed nationally,^25^ our finding may mirror population-level immunization changes rather than increased illness vulnerability among children with under-immunization.

## LIMITATIONS

Our study was limited by its retrospective nature, and we captured diagnoses coded by the emergency physician rather than by microbiology or imaging results that may have resulted after the ED visit. While influenza testing has rapid turnaround during an ED visit, respiratory viral polymerase chain reaction panels result after the ED visit in our facilities; therefore, the results would not have been available to inform the ED diagnosis. While only patients with health plan membership were included for data completeness, there were no major changes in health plan membership over the study period, and health plan members are similar to the surrounding communities demographically and in health status.^14^

## CONCLUSION

We evaluated the frequency of pediatric ED visits for acute respiratory illness in the pre-, pandemic, and post-vaccine periods, observing an initial flattening of ED visits followed by return to seasonal variation starting in 2022 with marked monthly surges in winter 2022. Future pandemic surge planning and mitigation strategies might consider the longer term impact of pandemic events on ED use, given that COVID-19 is now endemic and typical respiratory illnesses have resurfaced.

## Supplementary Information



## Figures and Tables

**Figure:**
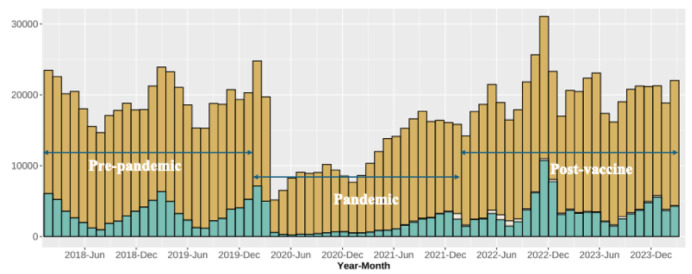
Monthly emergency department (ED) visits for acute respiratory illness, January 1, 2018–December 31, 2019 (pre-pandemic); January 1, 2020–December 31, 2021 (pandemic); and January 1, 2022–March 31, 2024 (post-vaccine); 0–17 years of age. Orange (top column): total ED visits that were not respiratory illnesses; beige (middle column): COVID-19 infection; green (bottom column): acute respiratory illness (non-COVID-19).

**Table 1:** Patient and visit characteristics of respiratory illness-related emergency department visits: pre-pandemic January 1, 2018–December 31, 2019; pandemic, January 1, 2020–December 31, 2021; and post-vaccine, January 1, 2022–March 31, 2024.

Characteristic	Overall N = 151,983^1^	Pre-pandemic: n = 49,912 (32.8%)^1^	Pandemic: n = 27,109 (17.8%)^1^	Post-vaccine: n = 74,962 (49.3%)^1^
Age				
< 28 days	1,653 (1.1)	490 (1.0)	391 (1.4)	772 (1.0)
28 days to < 3 months	3,885 (2.6)	1,106 (2.2)	815 (3.0)	1,964 (2.6)
3 months to < 2 years	43,323 (29)	14,366 (29)	7,993 (29)	20,964 (28)
2 years to < 5 years	43,341 (29)	13,696 (27)	7,803 (29)	21,842 (29)
5 years to < 11 years	38,272 (25)	12,458 (25)	5,984 (22)	19,830 (26)
11 years to <1. 8 years	21,509 (14)	7,796 (16)	4,123 (15)	9,590 (13)
Sex^2^				
Female	64,348 (42)	21,345 (43)	11,437 (42)	31,566 (42)
Male	87,620 (58)	28,562 (57)	15,670 (58)	43,388 (58)
Race and ethnicity				
American Indian/Alaskan Native	697 (0.5)	232 (0.5)	128 (0.5)	337 (0.4)
Asian	24,962 (16)	7,764 (16)	3,965 (15)	13,233 (18)
Black	19,721 (13)	7,396 (15)	3,777 (14)	8,548 (11)
Hispanic or Latino	53,195 (35)	17,228 (35)	9,373 (35)	26,594 (35)
Missing/Decline to state	11,895 (7.8)	3,273 (6.6)	2,097 (7.7)	6,525 (8.7)
Native Hawaiian or other Pacific Islander	2,692 (1.8)	943 (1.9)	486 (1.8)	1,263 (1.7)
White	38,821 (26)	13,076 (26)	7,283 (27)	18,462 (25)
Neighborhood Deprivation Index Quartile (1 = least deprived)^2^				
1	23,033 (15)	6,353 (13)	3,769 (14)	12,911 (17)
2	37,914 (25)	12,468 (25)	6,737 (25)	18,709 (25)
3	47,238 (31)	15,532 (31)	8,150 (30)	23,556 (31)
4	43,722 (29)	15,540 (31)	8,440 (31)	19,742 (26)
Unknown	76	19	13	44
Tobacco exposure				
Current or former	1,087 (0.7)	426 (0.9)	188 (0.7)	473 (0.6)
Never	128,702 (85)	42,327 (85)	21,642 (80)	64,733 (86)
Passive exposure	9,388 (6.2)	4,523 (9.1)	2,053 (7.6)	2,812 (3.8)
Unknown/missing	12,806 (8.4)	2,636 (5.3)	3,226 (12)	6,944 (9.3)
Under immunization	22,912 (15)	6,066 (12)	4,149 (15)	12,697 (17)
Respiratory condition from PE-CCS^2^	16,545 (11)	8,069 (16)	2,939 (11)	5,537 (7.5)
Tech-dependent condition from PE-CCS^2^	3,095 (2.1)	943 (1.9)	560 (2.1)	1,592 (2.1)
At least one complex chronic condition from PE-CCS^2^	33,633 (22)	13,246 (27)	5,970 (22)	14,417 (19)
ED length of stay^3^	2.00 (2.00)	2.00 (2.00)	2.00 (2.00)	2.00 (2.00)
ED disposition				
Admitted	7,108 (4.7)	2,210 (4.4)	1,375 (5.1)	3,523 (4.7)
Discharged	144,875 (95)	47,702 (96)	25,734 (95)	71,439 (95)

1 n (%).

2 missing < 1%.

3 Median (interquartile range).

*ED*, emergency department; *PE-CCS*, Pediatric Clinical Classification System.

**Table 2:** Most common emergency department visit diagnoses for each observation period: pre-pandemic January 1, 2018–December 31, 2019; pandemic, January 1, 2020–December 31, 2021; and post-vaccine, January 1, 2022–March 31, 2024.

Diagnosis category	Pre-pandemic n = 49,912 (32.8%)	Pandemic n = 27,109 (17.8%)	Post-vaccine n = 74,962 (49.3%)
Upper respiratory infection	15,143 (30%)	8,579 (32%)	24,613 (33%)
Asthma	7,508 (15%)	3,369 (12%)	9,302 (12%)
Cough	4,948 (9.9%)	3,300 (12%)	8,262 (11%)
Croup	6,097 (12%)	2,762 (10%)	7,457 (9.9%)
Influenza	5,949 (12%)	2,859 (11%)	7,029 (9.4%)
Pneumonia	3,793 (7.6%)	1,494 (5.5%)	4,400 (5.9%)
Bronchiolitis	3,032 (6.1%)	1,985 (7.3%)	4,618 (6.2%)
COVID-19 infection	0 (0%)	689 (2.5%)	4,907 (6.5%)
Shortness of breath	1,202 (2.4%)	1,093 (4.0%)	1,806 (2.4%)
Bronchitis	1,107 (2.2%)	390 (1.4%)	1,287 (1.7%)

Additional diagnoses (total of these diagnoses is 2.3%, 2.2% and 1.7%, of eligible visits for each period, respectively):

bronchospasm, chest pain, respiratory distress, acute respiratory failure, stridor, laryngitis, lower respiratory tract infection, pneumonitis, complicated pneumonia, acute tracheitis, and laryngotracheitis.
