# Supplementary material for: Respiratory Illness-related Emergency Visits Among Children, COVID-19 and Beyond: Observing a Return to Seasonal Patterns?
Source: West J Emerg Med. 2025 Dec 20;27(1):130–6. doi: 10.5811/westjem.46552 (PMC12815567; doi:10.5811/westjem.46552)
Supplement: Supplementary file 1 [file wjem-27-130-s001.docx]

Appendix: Included ICD-10 respiratory illness diagnoses (from PE-CCS respiratory illness diagnoses) ) (ref: <https://www.childrenshospitals.org/content/analytics/toolkit/pediatric-clinical-classification-system-peccs-codes>)

| **Diagnosis** | **ICD-10 code** |
| --- | --- |
| Acute URI | J06.9 |
| Croup | J05.0 |
| Cough, unspecified | R05.9 |
| Cough, unspecified | R05 |
| Influenza | J11.1 |
| Asthma, unspecified w acute exacerbation | J45.901 |
| Pneumonia | J18.9 |
| Bronchiolitis | J21.9 |
| COVID-19 disease | U07.1 |
| Influenza like illness | J11.1 |
| Asthma, unspecified | J45.909 |
| Bronchiolitis due to RSV | J21.0 |
| Shortness of breath | R06.02 |
| Reactive airway disease, unspecified | J45.909 |
| Wheezing | R06.2 |
| Nasal congestion | R09.81 |
| URI (upper respiratory infection) | J06.9 |
| Community acquired pneumonia | J18.9 |
| Bronchospasm | J98.01 |
| Bronchitis | J40 |
| Acute bronchitis due to RSV | J20.5 |
| Acute bronchitis | J20.9 |
| Dyspnea | R06.00 |
| RSV pneumonia | J12.1 |
| Acute URI (upper respiratory infection) due to RSV | J06.9 |
| Rib pain | R07.81 |
| Stridor | R06.1 |
| RSV bronchopneumonia | J12.1 |
| Respiratory distress | R06.00 |
| Acute cough | R05.1 |
| Common cold | J00 |
| Intermittent asthma | J45.20 |
| Influenza A | J10.1 |
| Nasal sinus congestion | R09.81 |
| Pleurisy | R09.1 |
| Asthma, unspecified w status asthmaticus | J45.902 |
| COVID-19 acute bronchitis | U07.1 |
| Acute respiratory failure | J96.00 |
| Acute hypoxemic respiratory failure | J96.01 |
| COVID-19 pneumonia | U07.1 |
| Laryngitis | J04.0 |
| Respiratory distress of child | R06.00 |
| Acute respiratory distress | R06.03 |
| Viral pneumonia | J12.9 |
| Influenza due to identified novel influenza A virus | J09.x2 |
| Pleuritic chest pain | R07.81 |
| Cough variant asthma | J45.991 |
| Atypical pneumonia | J18.9 |
| Mild persistent asthma | J45.30 |
| Acute viral bronchitis | J20.8 |
| Lobar pneumonia, LLL | J18.1 |
| Influenza B | J10.1 |
| Postviral cough | R05 |
| Postviral cough | R05.8 |
| Lobar pneumonia, RLL | J18.1 |
| Acute lower respiratory infection | J22 |
| Acute bronchospasm | J98.01 |
| Asthmatic bronchitis, unspecified | J45.909 |
| Lobar pneumonia, RML | J18.1 |
| Moderate persistent asthma | J45.40 |
| COVID-19 lower respiratory infection | U07.1 |
| Pertussis | A37.90 |
| Bacterial pneumonia | J15.9 |
| Nasopharyngitis | J00 |
| Chronic cough | R05 |
| COVID-19 multisystem inflammatory syndrome in children | U07.1 |
| Influenza pneumonia | J11.00 |
| Intermittent asthma, controlled | J45.20 |
| Pneumonitis | J18.9 |
| Acute nasopharyngitis due to RSV | J00 |
| Intermittent asthma w acute exacerbation | J45.21 |
| Acute lower respiratory infection due to RSV | J22 |
| Dyspnea on exertion | R06.09 |
| COVID-19 test positive by outside laboratory | U07.1 |
| Exercise induced asthma | J45.998 |
| Lobar pneumonia, RUL | J18.1 |
| Bronchopneumonia | J18.0 |
| Hypoxemic respiratory failure, unspecified acuity | J96.91 |
| Recurrent croup | J05.0 |
| Paroxysmal cough | R05 |
| Paroxysmal cough | R05.8 |
| Respiratory arrest | R09.2 |
| Bilat pneumonia | J18.9 |
| Influenza due to identified novel influenza A virus w upper respiratory symptoms | J09.x2 |
| Influenza w upper respiratory symptoms | J11.1 |
| Moderate persistent asthma w acute exacerbation | J45.41 |
| Respiratory bronchiolitis w interstitial lung disease | J84.115 |
| Avian influenza w upper respiratory symptoms | J09.x2 |
| Persistent cough | R05 |
| Severe persistent asthma | J45.50 |
| Lobar pneumonia | J18.1 |
| Lung abscess | J85.2 |
| Mild persistent asthma w acute exacerbation | J45.31 |
| Moderate persistent asthma, not controlled | J45.40 |
| Lobar pneumonia, LUL | J18.1 |
| Painful respirations | R07.1 |
| Acute tracheitis | J04.10 |
| Allergic asthma, unspecified | J45.909 |
| Allergic asthma, unspecified, w acute exacerbation | J45.901 |
| Asymptomatic COVID-19 disease | U07.1 |
| Cough syncope | R05 |
| Influenza w gastroenteritis | J11.2 |
| Mild persistent asthma, controlled | J45.30 |
| Seasonal asthma | J45.998 |
| Viral laryngitis | J04.0 |
| Acute bronchitis due to rhinovirus | J20.6 |
| Acute on chronic hypoxemic respiratory failure | J96.21 |
| Acute on chronic respiratory failure | J96.20 |
| Asthma, unspecified w allergic rhinitis w acute exacerbation | J45.901 |
| Bronchiectasis | J47.9 |
| Chest cold | J40 |
| COVID-19 acute respiratory distress syndrome | U07.1 |
| Lobar pneumonia, lingula | J18.1 |
| Mild persistent allergic asthma w acute exacerbation | J45.31 |
| Moderate persistent asthma, controlled | J45.40 |
| Pleural empyema | J86.9 |
| Severe persistent asthma w acute exacerbation | J45.51 |
| Subacute cough | R05.2 |
| Acute bronchitis due to parainfluenza | J20.4 |
| Acute hypercapnic respiratory failure | J96.02 |
| Acute laryngotracheitis | J04.2 |
| Bilat lower lobe pneumonia | J18.9 |
| Bordetella parapertussis infection | A37.10 |
| Bronchiolitis due to influenza virus | J11.1 |
| Bronchiolitis due to metapneumovirus | J21.1 |
| Influenza w otitis media | J11.83 |
| Intermittent asthma w status asthmaticus | J45.22 |
| Laryngotracheitis | J04.2 |
| Mild persistent asthma, not controlled | J45.30 |
| Mycoplasma pneumonia | J15.7 |
| Pseudomonas pneumonia | J15.1 |
| Tracheobronchitis | J40 |
| Acute adult respiratory distress syndrome | J80 |
| Acute bronchitis due to coxsackievirus | J20.3 |
| Acute bronchitis due to mycoplasma pneumoniae | J20.0 |
| Allergic asthma, unspecified w status asthmaticus | J45.902 |
| Avian influenza w pneumonia | J09.x1 |
| COVID-19 test positive by home test | U07.1 |
| Healthcare associated pneumonia. | J18.9 |
| Human metapneumovirus bronchopneumonia | J12.3 |
| Human metapneumovirus pneumonia | J12.3 |
| Infectious rhinitis | J00 |
| Influenza bronchopneumonia | J11.08 |
| Influenza due to identified novel influenza A virus w pneumonia | J09.x1 |
| Influenza w encephalopathy | J11.81 |
| Intermittent allergic asthma | J45.20 |
| Intermittent allergic asthma w acute exacerbation | J45.21 |
| Intermittent asthma w allergic rhinitis | J45.20 |
| Laryngotracheobronchitis | J40 |
| Lung abscess w pneumonia | J85.1 |
| Mild persistent asthma w allergic rhinitis w acute exacerbation | J45.31 |
| Moderate persistent asthma w allergic rhinitis w acute exacerbation | J45.41 |
| Parainfluenza bronchopneumonia | J12.2 |
| Parainfluenza pneumonia | J12.2 |
| Recurrent pneumonia | J18.9 |
| Respiratory failure, unspecified acuity | J96.90 |
| Severe persistent allergic asthma w acute exacerbation | J45.51 |
| Severe persistent asthma w status asthmaticus | J45.52 |
| Severe persistent asthma, not controlled | J45.50 |
